# Identification of Novel Bacteriophages with Therapeutic Potential That Target *Enterococcus faecalis*

**DOI:** 10.1128/IAI.00512-19

**Published:** 2019-10-18

**Authors:** M. Al-Zubidi, M. Widziolek, E. K. Court, A. F. Gains, R. E. Smith, K. Ansbro, A. Alrafaie, C. Evans, C. Murdoch, S. Mesnage, C. W. I. Douglas, A. Rawlinson, G. P. Stafford

**Affiliations:** a Integrated BioSciences, School of Clinical Dentistry, University of Sheffield, Sheffield, United Kingdom; b Department of Molecular Biology and Biotechnology, University of Sheffield, Sheffield, United Kingdom; c Department of Microbiology, Faculty of Biochemistry, Biophysics, and Biotechnology, Jagiellonian University, Cracow, Poland; d Department of Chemical and Biological Engineering, The University of Sheffield, Sheffield, United Kingdom; Georgia Institute of Technology School of Biological Sciences

**Keywords:** bacteriophage, oral microbiology, biofilm, capsule

## Abstract

The Gram-positive opportunistic pathogen Enterococcus faecalis is frequently responsible for nosocomial infections in humans and represents one of the most common bacteria isolated from recalcitrant endodontic (root canal) infections. E. faecalis is intrinsically resistant to several antibiotics routinely used in clinical settings (such as cephalosporins and aminoglycosides) and can acquire resistance to vancomycin (vancomycin-resistant enterococci). The resistance of E. faecalis to several classes of antibiotics and its capacity to form biofilms cause serious therapeutic problems. Here, we report the isolation of several bacteriophages that target E. faecalis strains isolated from the oral cavity of patients suffering root canal infections. All phages isolated were *Siphoviridae* with similar tail lengths (200 to 250 nm) and icosahedral heads. The genome sequences of three isolated phages were highly conserved with the exception of predicted tail protein genes that diverge in sequence, potentially reflecting the host range. The properties of the phage with the broadest host range (SHEF2) were further characterized. We show that this phage requires interaction with components of the major and variant region enterococcal polysaccharide antigen to engage in lytic infection. Finally, we explored the therapeutic potential of this phage and show that it can eradicate E. faecalis biofilms formed *in vitro* on a standard polystyrene surface but also on a cross-sectional tooth slice model of endodontic infection. We also show that SHEF2 cleared a lethal infection of zebrafish when applied in the circulation. We therefore propose that the phage described here could be used to treat a broad range of antibiotic-resistant E. faecalis infections.

## Abstract (precis)

The Gram-positive opportunistic pathogen Enterococcus faecalis is frequently responsible for nosocomial infections in humans and represents one of the most common bacteria isolated from recalcitrant endodontic (root canal) infections. E. faecalis is intrinsically resistant to several antibiotics routinely used in clinical settings (such as cephalosporins and aminoglycosides) and can acquire resistance to vancomycin (vancomycin-resistant enterococci).

## INTRODUCTION

Enterococcus faecalis is a common nosocomial pathogen that is frequently isolated from the bloodstream and wound infections (1, 2). In some cases, the clinical outcomes are poor due to the limited choices of effective antimicrobial therapy and colonization by vancomycin-resistant enterococci (VRE). One example is E. faecalis strain V583, which was the first of the vancomycin-resistant E. faecalis clinical isolates to be sequenced (3, 4). In addition, E. faecalis is commonly recovered from chronic periapical or root canal infections associated with failed endodontic therapy (5, 6). It has been proposed that this is associated with their ability to (i) live and survive in the presence of several commonly used root canal antiseptic irrigants (e.g., calcium hydroxide) (7), (ii) tolerate prolonged periods of starvation (8), (iii) form biofilms (9), and (iv) acquire antibiotic resistance (10, 11). Together, these features indicate that new therapeutic approaches are necessary.

Bacteriophage therapy has recently reemerged as an attractive alternative antimicrobial strategy to treat antibiotic-resistant biofilm forming pathogens. In some infections, such as those associated with burn wounds caused by Pseudomonas aeruginosa and Staphylococcus aureus, phage therapy is now considered an option for topical application (12). Furthermore, in the United States, the Food and Drug Administration recently approved a phage-based phase I clinical trial against P. aeruginosa, *Staphylococcus* spp. and Escherichia coli in chronic venous leg ulcers, illustrating that treatment using a phage cocktail was associated with no adverse reaction (13). For example, phase II clinical trials in Europe using phage therapy against chronic otitis externa caused by P. aeruginosa infection have been conducted with successful results (14). Excitingly, phages were also used intravenously in 2019 to cure a disseminated Mycobacterium abscessus infection in a 15-year-old patient in the United Kingdom (15).

E. faecalis lytic phages have been previously isolated using indicator strains of animal origin (16, 17), nonoral human isolates, and lab strains (18–21). Here, we set out to isolate bacteriophages targeting E. faecalis strains of oral origin, either directly from endodontic infections or from mouthwashes of patients receiving endodontic treatment and also from oral lesions sourced from a range of oral microbiology laboratories around Europe. A range of tailed phages were isolated from a wastewater treatment plant and characterized. We report their therapeutic potential against E. faecalis strains forming biofilms and their capacity to eradicate systemic infection in a zebrafish model of infection. Overall, our data further highlight that phages have great potential as therapeutic adjuncts in oral and other infections.

## RESULTS

### Isolation and characterization of bacteriophages targeting *E. faecalis*.

**FIG 1:**
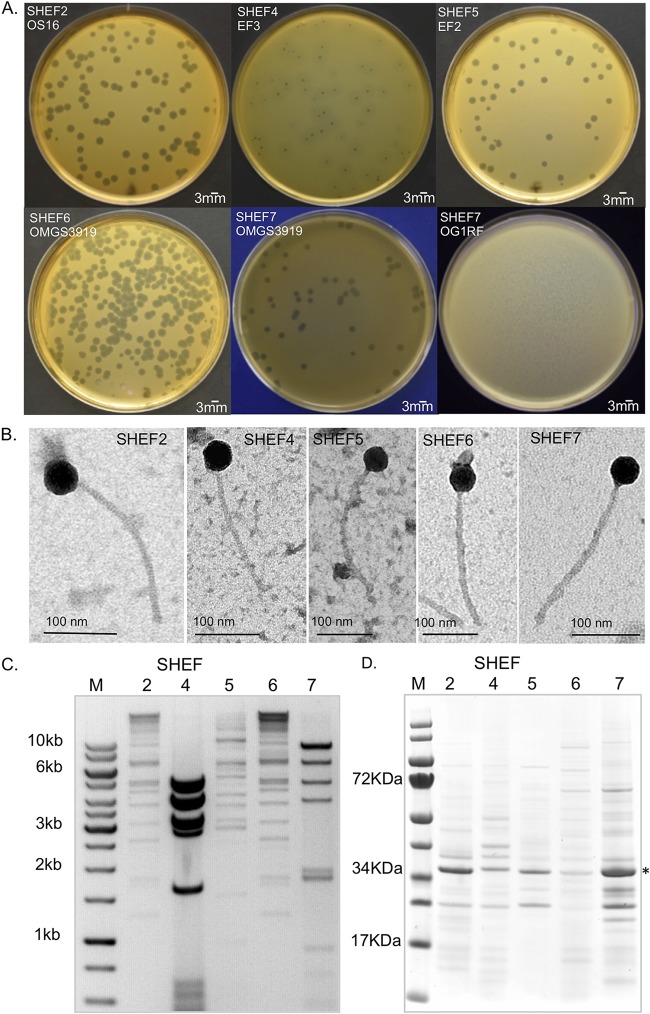
Isolation of E. faecalis bacteriophage and plaque morphology. (A) Images of bacterial plaques formed by the isolated phage in top-agar lawns of E. faecalis OS16 (SHEF2), E. faecalis EF3 (SHEF4), E. faecalis EF2 (SHEF5), and E. faecalis OMGS3919 (SHEF6 and -7). SHEF7 is also shown with E. faecalis OG1RF to illustrate plaque morphologies. (B) Transmission electron micrographs of SHEF phage particles. Phages were negatively stained with 0.2% uranyl acetate as described in Materials and Methods. Scale bars, 100 nm. (C) RFLP analysis of extracted phage chromosomal DNA. SHEF2, -4, -5, and -7 phage genomic DNA was digested with HindIII and analyzed by agarose gel electrophoresis (an inverted image is shown alongside the GeneRuler 1-kb ladder). (D) Virion protein profiles of SHEF2, -4, -5, -6, and -7 by SDS-PAGE with InstantBlue staining. The dominant protein band identified by MS/MS as the major capsid protein for SHEF2 at 36 kDa is indicated by an asterisk (*).

Sheffield wastewater was used as a potential source for E. faecalis-specific bacteriophages (several samples from independent sites). Phage plaques were first identified by spotting the processed sewage solution on top-agar lawns of a range of orally isolated clinical strains of E. faecalis (Table 1), with plaques successfully obtained with E. faecalis OS16, EF2, EF3, and OMGS3919. To isolate individual phages, this procedure was repeated twice and five bacteriophages—named SHEF2, -4, -5, -6, and -7—were identified using a range of E. faecalis isolates (Fig. 1).

**TABLE 1:** Bacterial strains used in the study

Bacterial strain	Source	Reference(s)
*E. faecalis*		
EF1, EF2, EF3, OS16	Oral rinse/endodontic patient	83, 84
ER3/2s	Oral orthograde retreatment	85
OMGS3197, OMGS3198	Oral endodontic strains	86
OMGS3885, OMGS3919	Oral mucosal lesions	86
EF54	Nonoral human isolate	51
OG1RF	Oral lab strain	87
OG1RF *epaB* TX5179; mutant harboring an insertion in *epaB* (formerly orfde4)		47
OG1RF *epaB* TX5179 + pTX5249	Complementation plasmid built and strain constructed by Zeng et al. (88)	88
OG1RF OPDV; OG1RF derivative with deletions in *oatA*, *pgdA*, *dltA*, and *sigV*	Constructed by Smith et al. (30)	30
OPDV_11720::Tn*2.5*; a transposon mutant harboring a mutation in OG1RF_11720	Isolated by Smith et al. (30)	30
OPDV_11720::Tn*2.5* + pTet-OG1RF_11720	Complementation plasmid built and strain constructed by Smith et al. (30)	30
OPDV_11715::Tn*2.13*; a transposon mutant harboring a mutation in OG1RF_11715	Isolated by Smith et al. (30)	30
OPDV_11715::Tn*2.13* + pTet-OG1RF_11715	Complementation plasmid built and strain constructed by Smith et al. (30)	30
OPDV_11714::Tn*2.14*; a transposon mutant harboring a mutation in OG1RF_11714	Isolated by Smith et al. (30)	30
OPDV_11714::Tn*2.14* + pTet-OG1RF_11714	Complementation plasmid built and strain constructed by Smith et al. (30)	30
OPDV_11707::Tn*2.8*; a transposon mutant harboring a mutation in OG1RF_11714	Isolated by Smith et al. (30)	30
OPDV_11707::Tn*2.8* + pTet-OG1RF_11707	Complementation plasmid built and strain constructed by Smith et al. (30)	30
JH2-2	Nonoral lab strain	89
		
*E. faecium* E1162	Clinical blood isolate; CC17	90

Three distinct plaque morphologies were identified, with SHEF2, -5, -6, and -7 phages forming plaques with 3 to 4 mm diameters surrounded by a thin area of secondary lysis of 1 mm, whereas SHEF4 formed 2-mm-diameter central plaques surrounded by halos of larger secondary lysis (Fig. 1A). When infecting the strain OG1RF, SHEF7 formed small pin-hole-sized plaques 1 mm in diameter without distinct secondary lysis, whereas it formed large plaques with OMGS3919 (Fig. 1A).

Negative staining transmission electron microscopy of purified phages revealed that all bacteriophages had polyhedral head shapes and noncontractile long tails ranging from 200 to 250 nm and polyhedral heads (Fig. 1B) with diameters between 41 and 46 nm (Table 2). According to the 2005 guidelines of the International Committee on Taxonomy of Viruses, the SHEF bacteriophages were classified as belonging to the family *Siphoviridae* (order *Caudovirales*) based upon their tail morphology (22).

**TABLE 2:** Head and tail dimensions of isolated E. faecalis phages

Phage	Mean ± SD^*a*^	
	Head diam (nm)	Tail length (nm)
SHEF2	42.34 ± 1.0	231 ± 1.3
SHEF4	45.60 ± 1.0	199.4 ± 0.8
SHEF5	44.32 ± 0.9	240.5 ± 1.5
SHEF6	45.81 ± 0.4	250.6 ± 3.0
SHEF7	41 ± 0.1	230 ± 2.4

a At least three phage particles were measured for each phage type, and the mean values were used to calculate the dimensions.

### Molecular characterization of isolated phages.

**FIG 2:**
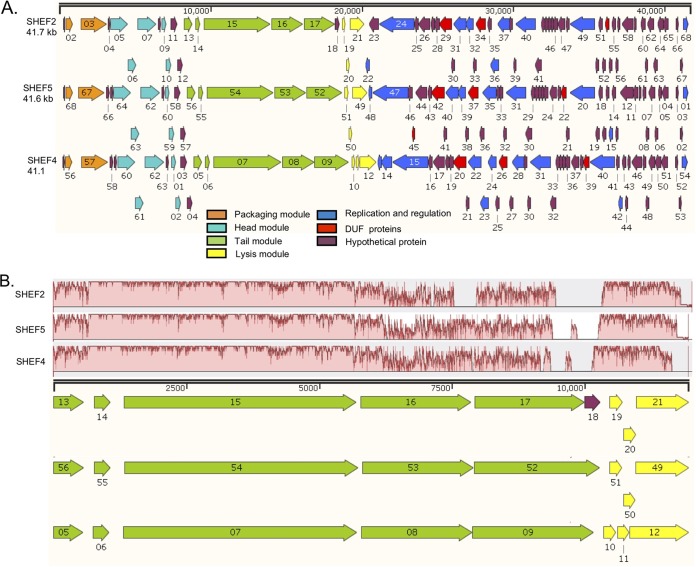
Genome organization of E. faecalis lytic phages SHEF2, SHEF5, and SHEF4. (A) Images produced using SnapGene Viewer 1.1.3 software (DUF, conserved domains of unknown function). Genome annotation corresponds to GenBank accession numbers MF678788, MF678789, and MF678790 for SHEF2, -4, and -5, respectively. The colors correspond to the predicted function, as indicated in the key. (B) Mauve alignment of tail and lysis genes highlighting areas of conservation.

Digestion of phage DNA with restriction enzymes and DNase I indicated that all isolated phages were double-stranded DNA viruses. Restriction fragment length polymorphism (RFLP) tests were performed on phage chromosomal DNAs (Fig. 1C). The restriction profiles of SHEF2 and -6 were very similar, whereas all the others were different. From the RFLP analyses, the five phage genome sizes were estimated to be in the range of 39 to 43 kbp. Next, we analyzed purified phage particles by SDS-PAGE (Fig. 1D). Distinct protein profiles suggested five separate phages were isolated. All samples revealed a prominent band at 36 kDa (Fig. 1D), which mass spectrometry (MS) confirmed as the phage major capsid protein for SHEF2 (Fig. 2, SHEF2_07). Although it is likely that this band is the head protein in all cases, we do not have MS data to confirm this. In short, we successfully confirmed the isolation of five separate E. faecalis bacteriophages of the family *Siphoviridae*.

### Determination of host range.

The host range of the phages isolated was studied using both spot and soft agar overlay (SAO) methods at a multiplicity of infection (MOI) of 0.1 to detect visible plaques. All five SHEF bacteriophages were specific to E. faecalis since none could produce visible plaques using E. faecium (data not shown). Host range tests showed that all phages have distinct strain specificity preferences, with SHEF2 displaying the broadest host range with the capacity to lyse 9 of the 13 E. faecalis indicator strains tested, followed by SHEF6 and -7. SHEF5 and -4 possess the narrowest host ranges, lysing only three and two strains, respectively. Despite the similarities between SHEF2 and -6 at the genomic level, their host ranges are not identical, indicating that they are distinct phages. No relationship between host range and multilocus sequence type (MLST) was found.

### Genome organization of SHEF2, -4, and -5.

The genomes of phages SHEF2, -4, and -5 revealed similar sizes of 41.7, 41.1, and 41.6 kbp, respectively, and appeared to be organized into two halves, transcribed in opposite directions (Fig. 2A). Each genome was assembled into one large contig with low read mapping coverage at the 5′ and 3′ ends and no clear edges at the ends of the contigs, suggesting circularity of terminally redundant permuted genomes. Four gene clusters corresponding to DNA packaging, structural components, cell lysis, and regulation and replication were identified (Fig. 2). There is little noncoding DNA between these genes, suggesting single transcripts for each set of genes. Although each phage exhibits a different host range, a high identity was found for both DNA and primary amino acid level of between 77 and 94% (see Fig. S1). Importantly, no putative integrase encoding genes were detected in the genome sequences, suggesting that the SHEF phages are likely to be lytic in nature.

All SHEF phages share a similar distribution pattern for DNA packaging and head morphogenesis genes (Fig. 2A, orange) and are upstream of the terminase (23, 24). The predicted head module (Fig. 2A, cyan) harbors genes encoding portal proteins (for genome injection into host cells), prohead protease maturation, head capsid proteins, and head-tail adaptor proteins. These genes are followed by tail and tape measure proteins (Fig. 2A, green) and a lysis module (Fig. 2A, yellow) containing a putative hemolysin XhlA, putative holin, and endolysin genes. SHEF2 and -5 encode putative endolysins (SHEF2_21 and SHEF5_49) with an N-terminal Amidase_2 domain and a predicted C-terminal ZoocinA_TRD (pfam16775) domain. SHEF4 encodes a putative endolysin with the same N-terminal domain but an SH3b peptidoglycan-binding domain, indicating differing cell wall targeting mechanisms.

The replication and regulation modules are also clustered and ordered identically, except that SHEF4 encodes an adenine-specific methyltransferase (modification methylase DpnIIB) that is absent from SHEF5. In addition, SHEF4 and -5 harbored an additional putative transcriptional regulator gene that is absent from SHEF2, and this suggests that all three employ slightly different modes of postreplication and DNA modification that might be crucial during the infection cycle.

Phage tail proteins are involved in the primary recognition and adsorption to specific receptors on the bacterial cell surface (25, 26). Since SHEF2, -4, and -5 exhibit different host ranges, we examined the amino acid sequence of the predicted tail proteins (Fig. 2B). The first three proteins displayed a high sequence similarity (81 to 100%). In contrast, the N-terminal 130 amino acids (aa) (out of 695 aa) of the fourth tail protein (SHEF2_16, SHEF4_08, and SHEF5_53), shares very high similarity (>95%) across our phage, while the rest of the sequence was highly divergent (see Fig. S2A in the supplemental material). We obtained similar results with the fifth tail protein (SHEF2_17, SHEF4_09, and SHEF5_52), where the first 175 aa (out of 800 aa) encodes conserved putative tail domains (TIGR01665 and pfam06605), before the rest of the protein sequence diverges (Fig. S2B). We propose that these two genes may be key to the host range determination of these phages during infection, as has been seen for other E. faecalis phages (27), and represent alternate cell wall binding proteins and domains.

### Identification of enterococcal polysaccharide antigen as the SHEF2 receptor.

**FIG 3:**
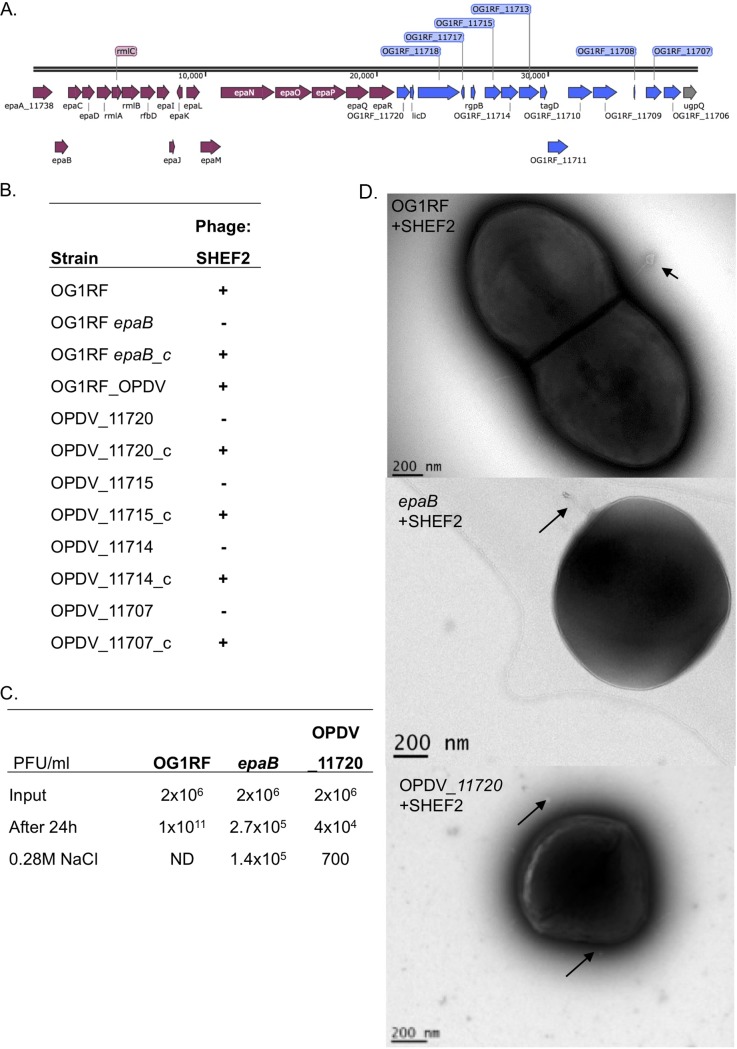
Molecular determination of SHEF2 phage adhesion using strain OG1RF. (A) Schematic of the OG1RF *epa* core (purple) and variable locus (blue), generated using SnapGene (labeling is according to accession number NC_017316.1). (B) Table showing qualitative results of spot assay double-layer agar infections of strains listed with SHEF2 (+, infection; –, no infection) (for pictures, see Fig. S4). (C) Phage adsorption assay for OG1RF and its isogenic *epaB* and *OPDV_11720* mutant strains (all at 10^8^ CFU/ml bacteria) with SHEF2. Phages (input, 2 × 10^6^) were added for 24 h before the phages were enumerated in cell supernatants before and after treatment with 0.28 M NaCl using a titer assay. ND, not determined (due to all cells being dead). This experiment was repeated twice, in triplicate each time (the mean is shown), with one example displayed here. (D) TEM images of infected OG1RF, *epaB*, and *OPDV_11720* mutants with SHEF2 at 30 min postinfection. Arrows indicate adsorbed phage.

To gain further insight into the mechanisms driving strain specificity in these phages, we sought to identify the receptor for SHEF2. We considered a candidate to be the prominent cell surface rhamnopolysaccharide enterococcal polysaccharide antigen (EPA) (27–29). The genetic locus encoding the synthesis of EPA is composed of 18 highly conserved genes (*epaA* to *epaR*), followed by a variable region that is divergent between strains (Fig. 3A) (30) and which has been proposed to encode strain-specific decoration of the core polysaccharide synthesized by a conserved genetic region (30, 31). Disruption of *epaB* [strain TX5179(*epaB*)], a key rhamnosyl transferase involved in EPA backbone synthesis, abolished the infectivity of SHEF2, with infectivity restored upon complementation with a plasmid containing the *epaBCD* operon (Fig. 3B). We then tested infection of an *oatA pgdA dltA sigV* strain, characterized by Smith et al. (30), which has altered peptidoglycan and teichoic acid production, showing that it is still sensitive to infection and that these molecules are not receptors for SHEF2.

As mentioned above, the core EPA is further decorated by variable chemical modifications that modulate virulence. We next tested the infectivity of SHEF2 against mutants in the *epa* variable region using mutants previously shown to impair EPA decorations and virulence (30). Three of the mutants had insertions in genes encoding glycosyltransferases (*OPDV_11720*::Tn, *OPDV_11715*::Tn, and *OPDV_11714*::Tn), and one had an insertion in an epimerase gene (*OPDV_11707*::Tn). All variable-region mutants showed a markedly reduced infectivity by SHEF2, which was restored upon in *trans* complementation. Interestingly, the *OG1RF_11714* mutant was still partially infected by SHEF2, with a slightly opaque plaque observed, that was restored to a typical clear plaque by complementation (Fig. 3B and S4).

Despite this lack of infection of *epa* mutant strains, it was still possible that binding occurred but that the phage was unable to complete its full lytic cycle. To explore this possibility, we carried out an adsorption assay. In this assay, phages and bacteria were incubated together for 24 h to allow the adsorption of phages or infection to proceed before the addition of 0.28 M NaCl, a treatment known to interfere with electrostatic phage-bacterium interactions but not harm *Enterococcus* cell viability. For the TX5179(*epaB*) strain the total phage numbers did not increase over 24 h, again indicating a lack of infection and expansion compared to the OG1RF strain (Fig. 3C). However, the NaCl treatment released 1.4 × 10^5^ PFU/ml of phage (51.8%, *P* < 0.01 versus OG1RF, _11720), reflecting weak adsorption of phages that do not enter a lytic life cycle but are still viable. Adsorption without population increase was also observed with the *OPDV_11720*::Tn*2.5* strain; however, fewer phages were recovered after 0.28 M NaCl treatment (1.75%, *P* < 0.01 versus OG1RF, *epaB*), indicating a stronger interaction with more phages left adsorbed after NaCl treatment. To exclude the possibility that phage progeny were trapped inside the host cells that could not lyse the host membrane and escape, the cell suspensions were treated with chloroform, with no further release of viable phage particles. Finally, to examine qualitatively that the released phage is bound to the cells, electron microscopy was performed on OG1RF, TX5179(*epaB*), and *OPDV_11720*::Tn*2.5* cells cultured with phages. Indeed, phages could be observed on all these strains (Fig. 3D), indicating that cell surface binding is still occurring in these mutants and that the core and variable EPA are both bound by phage SHEF2. Like others (27), we observed rounded cell morphology in TX5179(*epaB*) (Fig. 3D) and also with the *OPDV_11720*::Tn*2.5* strain.

### Infection parameters and strain preference of SHEF2.

**FIG 4:**
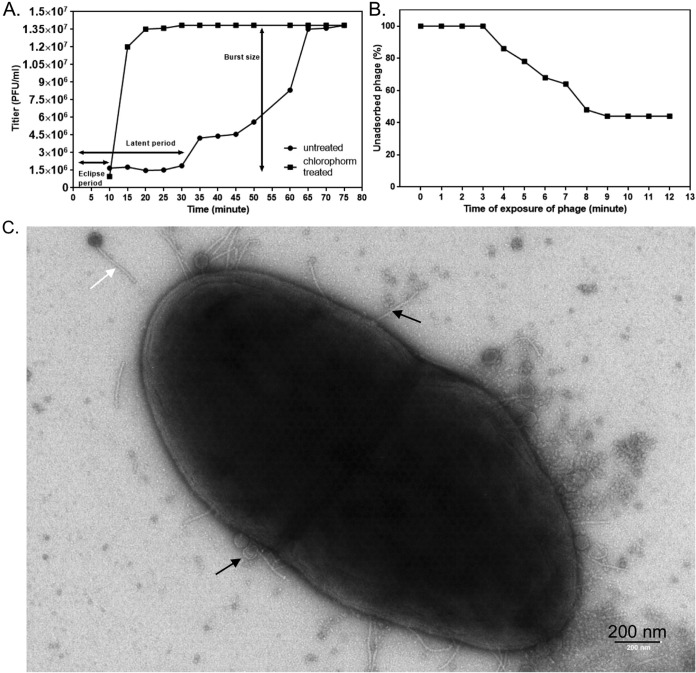
One-step growth and adsorption rate characterization of SHEF2. (A) One-step growth curve of SHEF2 phage with E. faecalis OS16 as the host. The two sets of data represent samples treated with or without chloroform. The eclipse, burst, and latent periods are labeled. (B) Adsorption of SHEF2 phage to E. faecalis OS16 expressed as a percentage of the total phages added. (C) Transmission electron micrograph of strain OS16 + SHEF2 at 30 min postinfection. Black arrows, spent heads and adsorbed phage; white arrow, unadsorbed phage.

We further characterized the host range and infection characteristics of bacteriophage SHEF2. As shown in Fig. S5A in the supplemental material, strain OS16 (as well as ER3/2s and EF54) was highly sensitive to SHEF2, as assessed by time course infection experiments, with cultures lysing within the first 60 min, compared to strains such as OMGS3197, OMGS3919, or OMGS3198, which lyse in the midexponential phase (2 h), and V583, which lyses in the late exponential phase (3 h) (Fig. S5). As a result and since it is an orally isolated clinical strain, we used strain OS16 to perform a one-step growth experiment to establish the eclipse period (i.e., the average time to produce the first mature intracellular phage), latent period (the average time to cell lysis), and burst size (the average number of phages released at cell lysis), which showed SHEF2 to be a highly efficient E. faecalis-targeting phage, with an eclipse period of only 10 min, a latent period of only 30 min (Fig. 4A), and a burst size of 9.3 PFU for this strain. The plateau phase was reached after 75 min, after a 45-min burst period. Next, we examined the adsorption parameters of this phage with the E. faecalis strain OS16 and found that saturation of adsorption was reached after 10 min (Fig. 4B). This adsorption is illustrated in Fig. 4C where a transmission electron microscopy (TEM) image taken of cells at 30 min postinfection illustrates phages attached to the cell surface.

### Ability of SHEF2 to clear *E. faecalis* biofilms.

**FIG 5:**
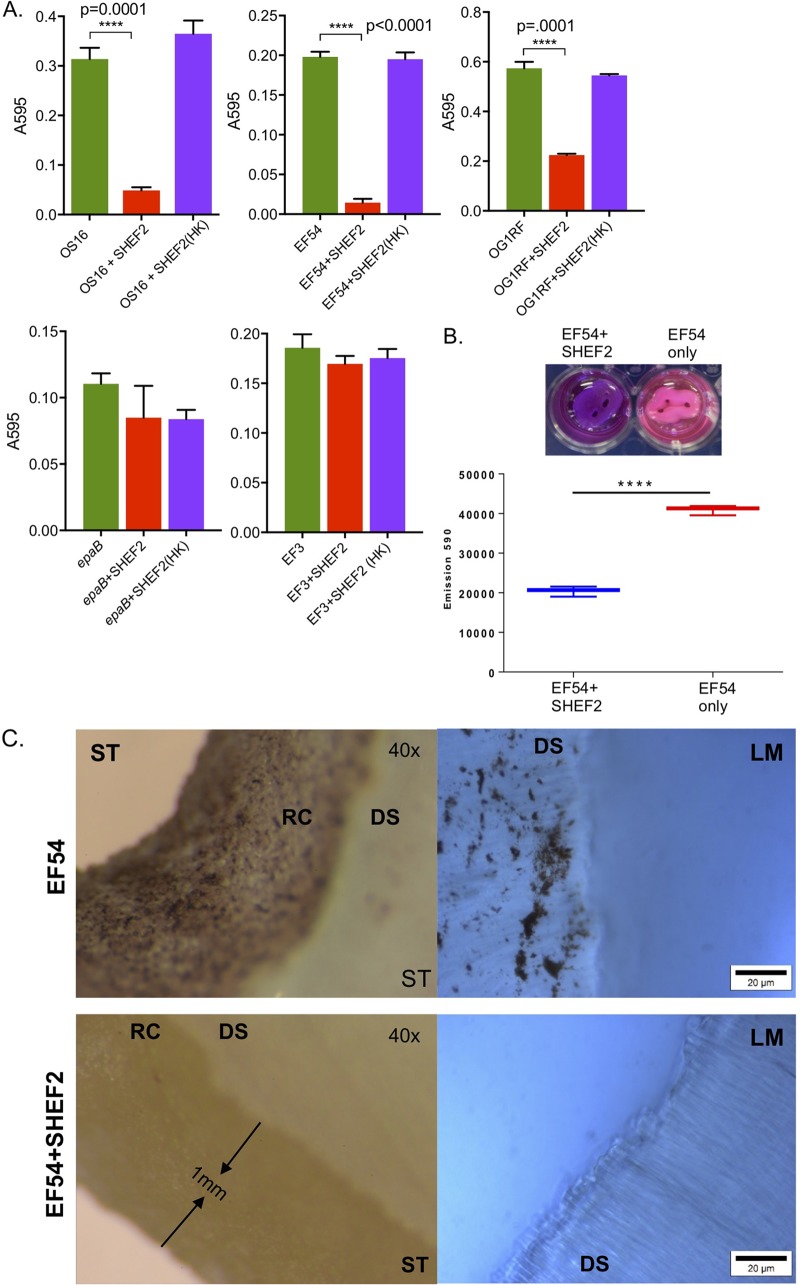
Bioﬁlm assay on polystyrene plates and tooth root slices. (A) Bar charts represent biofilm growth, as measured by crystal violet staining (measured using the *A*_570_ of the extracted stain, normalized to the cell growth in each well [*A*_600_]). Samples treated with live phage are labeled “+SHEF2,” while those with heat-killed [+SHEF2 (HK)] strain names are as shown elsewhere. Means of six polystyrene microtiter wells per condition are shown along with the SD, and a Student *t* test was used to compare conditions (*P* < 0.0001). Experiments were conducted on three separate occasions; one example is shown here. (B, upper panel) Photograph of tooth root slices *in situ* treated with SHEF2 (+SHEF2) or with strain EF54 only. The difference in color represents the resazurin reduction in response to resorufin and is represented quantitatively below (mean, SD from three readings). A Student *t* test was used to compare treated and untreated groups (*P* < 0.0001). (C) Stereomicroscope (ST, left) and light microscope (LM, right [resazurin stained]) images represent the untreated group (upper), while the lower images show SHEF2-treated samples. Biofilms of E. faecalis colonies scattered on the root canal surface (RC) and the dentinal surface (DS) of the ST and LM images, respectively, are shown.

Given that most bacteria in nature and in clinical infections reside in biofilms (32, 33), we tested the ability of SHEF2 to eradicate E. faecalis biofilms *in vitro* on inert polystyrene surfaces, using a range of strains. As seen in Fig. 5A, we tested the phage against 24-h preformed biofilms using heat-killed phage as a negative control and demonstrated phage-dependent clearance to various degrees (3- to 10-fold) for all sensitive strains (EF54, OS16, and OG1RF), whereas we observed no clearance for strains EF3 and OG1RF (*epaB*), which were insensitive to phage infection in plate-based and broth-based assays. In addition, we tested the clearance of biofilms of strains EF54 and OS16, as well as a mixed strain biofilm that had been preformed for 6 days (Fig. S3A and B), again showing clearance of biofilm in a phage-dependent manner.

We next tested the efficacy of phage SHEF2 to eradicate biofilms on natural tooth root surfaces by growing EF54 biofilms for 168 h before adding SHEF2 (10^8^ PFU/ml) for 3 h. We achieved a significant reduction in bacterial numbers, as indicated by a reduction in detectable metabolic activity (Fig. 5B; *P* < 0.0001 [resazurin assay]) to approximately 7 × 10^3^ bacteria, estimated using standard curves of emission (at 590 nm) versus CFU/ml (Fig. S3C) from an original input of more than 10^6^. (We were unable to do this on more strains due to limitations of available tooth slices and ethics restrictions.) We also observed, qualitatively, a drastic decrease in bacterial material via reduction in visible bacterial biomass visualized as the dark material in this image using stereomicroscopy and light microscopy (Fig. 5C).

### Phage treatment evaluation in a zebrafish model of infection.

**FIG 6:**
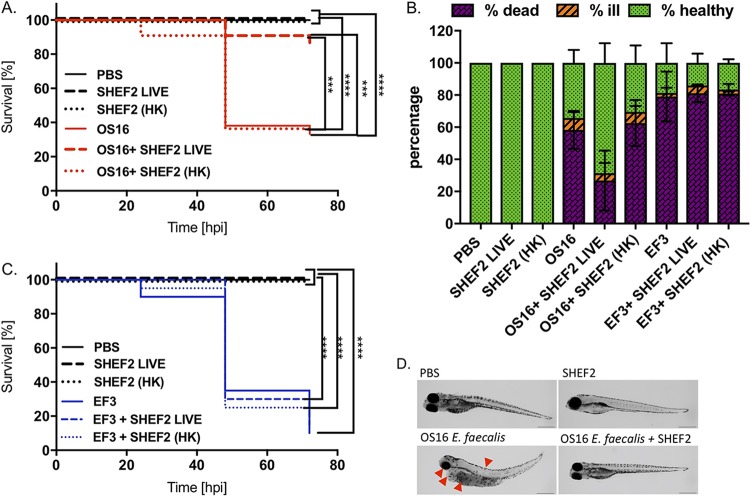
Phage SHEF2 treatment of E. faecalis OS16-infected zebrafish embryos. Zebrafish were infected systematically with E. faecalis OS16 strain or strain EF3 at a dose of 30,000 CFU at 30 h postfertilization. After 2 h, the embryos were injected with SHEF2 (SHEF2 LIVE) phage or heat-inactivated [SHEF2(HK)] at an MOI of 20 (in 2 nl). Control experiments were also performed with phage only [SHEF2 LIVE or SHEF2(HK)] and PBS (2 nl). Data are presented as Kaplan-Meier survival plots (A and C), as well as a bar chart (B), indicating mortality data at 72 hpi or all conditions. Bars represent means ± the SD. Three independent experiments were performed (*n* = 20 zebrafish per condition per experiment). Statistical comparison between groups was performed using a log-rank test (A and C) or one-way ANOVA (B). (D) Morphology of zebrafish embryos at 72 hpi after injection with PBS, E. faecalis OS16 alone, SHEF2 LIVE alone, or E. faecalis OS16, followed by SHEF2 LIVE (D). Red arrows indicate symptoms of infection (eye and yolk sac abnormalities, spinal curving, and pericardial edema). Scale bar, 500 μm.

Next, we performed systemic infection studies using an established zebrafish model of infection (34) and the clinical strain OS16. Zebrafish embryos were infected with E. faecalis for 2 h before being injected with SHEF2 or a heat-killed sample of SHEF2 at an MOI of 20 (with respect to the E. faecalis inoculum), alongside virus-only controls. Fish mortality and health status were then monitored for up to 72 h postinfection (hpi). E. faecalis OS16 caused a time-dependent lethality that was significantly higher (*P* < 0.0001) than the result for the phosphate-buffered saline (PBS) control or phage alone (Fig. 6A and B). Although injection with heat-killed SHEF2 (HK-SHEF2) phage postinfection with strain OS16 did not improve mortality rates of the zebrafish (73% dead, identical to OS16 only), injection of live SHEF2 resulted in only 16% death (and thus 84% survival) (*P* < 0.0001 versus OS16 only). Of note, all fish injected with phage only (SHEF2 LIVE) or inactivated phage only [SHEF2(HK)] were healthy. The morphology and overall health status of the fish were also monitored, showing that viable phage allowed recovery from OS16 infection, whereas the SHEF2(HK) did not (Fig. 6B). We also infected embryos with strain EF3, which is not sensitive to SHEF2 infection (Table 2) but still displayed the ability to cause mortality in embryos by 72 hpi (90%, *P* < 0.0001) compared to PBS-injected, live-phage-injected, or killed-phage-alone-injected fish (Fig. 6B and C). Significantly, neither viable nor heat-killed SHEF2 phage caused an improvement in survival when injected alongside strain EF3, with 81.13% ± 5.46% and 80.13% ± 1.27% death, respectively. In all experiments, the embryos infected with E. faecalis only displayed a lack of circulation, yolk sac, and eye abnormalities, alongside pericardiac edema and spine curvature (Fig. 6D, shown for OS16). In contrast, the majority of phage-treated zebrafish remained healthy throughout the experiment, with a health status comparable to that of the phage-only and PBS-injected controls (Fig. 6B).

## DISCUSSION

In this paper we report the isolation of five lytic phages isolated from wastewater using a range of oral and nonoral E. faecalis strains. Surprisingly, we were unable to isolate phages from oral samples against our strains, even though we could visualize phages in concentrated saliva (not shown) and even though metagenomic evidence from others showed that the oral cavity is rich in bacteriophage DNA (35).

All of the phages described in this study (SHEF2, -4, -5, -6, and -7) belonged to the family *Siphoviridae*, a family of phages previously found to target E. faecalis (16, 19, 36, 37), alongside members of the *Myoviridae* (18, 20, 21).

Our isolated phages (SHEF2, -4, -5, -6, and -7) possessed different host ranges, with SHEF2 having the broadest. After genome sequencing, we found strong similarity between the genome sequences of SHEF2, -4, and -5 (SHEF2 is 92 to 94% identical to SHEF4 and -5, respectively) but significant divergence in the putative tail gene locus genes (SHEF2_16, SHEF2_17, SHEF4_08, SHEF4_09, SHEF5_53, and SHEF5_52) with evidence of conserved N-terminal domains but different putative bacterial surface receptor binding domains in the C termini of these proteins. Similar observations have been made in various species, including Lactococcus lactis phage (38, 39) and *Streptococcus thermo lus* phage DT1, where interchange of the C-terminal domain of ORF18 with that of another phage (MD4) altered the host range specificity (40). We suggest that tail module genes 4 and 5 encode the host range specificity, determining the tail proteins that contain potentially novel E. faecalis surface binding domains. These findings will open the way for further investigation of phage host specificity and the adhesion of Gram-positive bacteria.

To probe the cell surface receptor for the SHEF2 phage, we showed that it was unable to productively infect insertion mutants with altered core EPA (*epaB*) or EPA decoration genes. All decoration mutants showed reduction in infectivity, except for the *OPDV_11714*::Tn*2.14* mutant. The partial infectivity of the *OPDV_11714*::Tn*2.14* mutant is in line with previous observations showing that mutation of this gene has only a small impact on EPA decoration (30). For both the *epaB* and the *OPDV_11720* (decoration) strains, the lack of infectivity is despite the phage retaining the ability to adsorb to the bacterial cell surface. Interestingly there is a discrepancy between the numbers of phages recovered from the cell surfaces of TX5179 and *OPDV_11720*::Tn*2.5* strains, with more phages still bound to the decoration-deficient mutant. These data suggest that SHEF2 likely binds to both the core polysaccharide and decoration residues and needs both for infection. In a similar manner, this is also the case for several well-characterized bacteriophages, e.g., Escherichia coli T4 phage (which requires lipopolysaccharide and OmpC binding) (41) or S. aureus phage 3C, that require teichoic and peptidoglycan for irreversible binding (25, 42).

For phages infecting Gram-positive organisms, the injection of viral DNA requires crossing the peptidoglycan layer and interaction with the cell membrane. This process has been studied in L. lactis phages that bind to rhamnose moieties of the cell wall before engaging the plasma membrane of the host (43–45). This process often employs phage-encoded glycosidases, or lysozyme-like enzymes (25), with all of the sequenced SHEF phages also encoding a tail protein with a putative lysozyme domain (SHEF2_15, SHEF4_07, and SHEF5_54). Evidence for two-stage adsorption and infection of E. faecalis exists in the case of E. faecalis and phage VPE25 infection, where a plasma membrane protein (EF0858) seemed to be required for lytic infection (and hence DNA injection) but not phage adsorption to the cell surface receptor (46). We therefore postulate here that the EPA and its variant decoration are required for productive binding of SHEF2 to the cell surface of E. faecalis with EPA and an unknown molecule acting as coreceptor for this phage. Based on the evidence in the literature (47, 48) that the EPA polysaccharide (PS) is not detectable on the outer surface of E. faecalis, it is tempting to speculate that SHEF2 initially binds first to an outer cell surface PS, followed by EPA, but we have no evidence for this. Overall, further tests are required to establish the primary receptor of SHEF2 interaction, potentially by producing recombinant versions of the tail protein domains and investigating their sugar-binding properties. Such studies will open new insight into possible resistance mechanisms for phage infection but also new targets for anti-infectives against E. faecalis or strain-specific diagnostics based on cell wall component binding capability.

The capacity to form biofilms is critical for E. faecalis virulence, providing resistance to antibiotics and allowing infections to persist. Studies have shown a strong association between virulence and biofilm formation, with 15 to 80% of clinical isolates being classified as strong biofilm formers (49–51). In addition, 75% of enterococcal infections in humans (bloodstream, urinary tract, and wound infections) are caused by E. faecalis (2). We showed that SHEF2 can significantly reduce biofilm formation of a range of sensitive E. faecalis strains (3- to 10-fold) that were preformed (24 h) on polystyrene surfaces (mimicking catheters for example), as well as on a novel *in vitro* tooth cross-section biofilm model (i.e., human tooth cross-sections). We believe this to be a strong indication that phage therapy based on SHEF2 and potentially our other phages might be useful for the eradication of root canal infections. In support of our data, an *ex vivo* root canal model developed by Khalifa et al. (20) was also significantly cleared of bacteria by phage. The use of bacteriophages therefore appears to be a promising strategy to reduce the biofilm bacterial load associated with E. faecalis infections, providing a potential adjunctive therapy for root canal infection that has failed to respond to conventional treatment. Other oral infections, such as periodontal disease, which is a complex mixed-species infection, would require phages targeting other oral pathogenic bacteria. However, based on reports of phages targeting Aggregatibacter actinomycetemcomitans (52) and *Fusobacterium* spp. (53, 54), as well as several *Streptococcus* (55), *Veillonella* (56), and *Neisseria* (57) spp., in the literature, this may be a feasible future approach.

In addition to oral infections, E. faecalis is a well-known cause of septicemia (58), with reports showing that oral bacteria can enter the bloodstream and disseminate systemically, contributing to infections such as endocarditis and rheumatoid arthritis (58, 59). We tested the therapeutic potential of the phages we isolated in a well-established *in vivo* zebrafish embryo systemic infection model. We showed, for the first time, that systemic phage treatment after infection with E. faecalis dramatically decreased the mortality of zebrafish embryos and greatly improved their health during infection, indicating the potential of this phage in treating systemic E. faecalis infections. It is important to note that neither the phage nor the bacterial components released upon lysis displayed toxicity toward the embryo, further demonstrating the potential for safe use of the phage systemically. The zebrafish infection model also served extremely well as a system to test the efficacy of phages against systemic bacteria and acts as a powerful tool for monitoring the dynamics of infection and phage clearance of infection that can be monitored in real time (60). Given the effectiveness of the phages described in killing planktonic and biofilm-associated E. faecalis, as well as in a systemic infection model, their therapeutic use could be extended to other infection types, such as sepsis, wound infections, or urinary tract infections.

It is well documented that bacteria can gain resistance to bacteriophages, and indeed resistance to E. faecalis phage has been reported (61). Although we did not isolate resistant strains during our experiments, we cannot rule out that this could occur and consider that the use of a cocktail of phage targeting different cellular receptors would be the best mode to reduce and combat resistance arising. Of note here is that resistance arising to at least phage SHEF2, for example, would require alterations in the EPA core or variable moieties, an alteration that would likely result in reduced virulence (30). It is worth noting here that many of the strains that were sensitive to our small panel of phages in this study were resistant to a range of antibiotics, including vancomycin (e.g., strain V583), illustrating that phages have the potential to be used as an adjunct or alternative treatment in infections caused by antibiotic-resistant strains of important human pathogens.

In conclusion, this study highlights isolation of phages targeting E. faecalis strains, in particular targeting a major virulence determinant of these strains (EPA), and establishes their potential use in treating biofilm infections by testing them in two clinically relevant model infection systems. Our work thus strengthens the possibility of developing phages as therapeutics to combat hard-to-treat oral, topical, and systemic infections.

## MATERIALS AND METHODS

### Bacterial strains used for bacteriophage screening.

The bacterial strains used in these investigations and their sources are listed in Table 1 (30). *Enterococcus* strains were grown aerobically with 5% CO_2_ at 37°C on brain heart infusion (BHI) agar (Oxoid, UK).

### Bacteriophage isolation.

Bacteriophages were isolated from wastewater collected from the inlet of a water treatment plant in the Sheffield area (United Kingdom) that treats both industrial and domestic wastewater. The water was filtered through 3M filter paper on site to remove particles but was not treated chemically or biologically. Samples were immediately brought to the laboratory and centrifuged at 7,000 × *g* for 15 min to remove remaining debris. The resulting supernatant was then passed through a 0.45-μm-pore-size filter (Sartorius, Germany) before 200 ml of sample was further centrifuged at 35,000 × *g* for 90 min to pellet any phage particles. The pellets were carefully resuspended overnight in 2 ml of SM buffer (1 M Tris-HCl buffer [pH 7.4] with 5 M NaCl, 1 M MgSO_4_, and 1% gelatin) at 4°C. Then, 10 μl of the suspended sample was spotted onto a double-layer agar plate. The bottom agar was composed of BHI solid agar supplemented with 5% (vol/vol) horse serum (Oxoid) and was overlaid with 3 to 4 ml of molten BHI soft agar (0.7%) containing 200 μl of test bacterial strain (overnight culture inoculum, optical density at 600 nm [OD_600_] of ∼2). Any formed plaques were picked using a sterile Pasteur pipette, deposited in 1 ml of SM buffer, and incubated overnight at 4°C before filtering through a 0.45-μm-pore-size syringe filter. Isolated phages were then expanded by infection using 100 μl of this sample with 200 μl of exponential-growth-phase indicator bacteria for 10 min before mixing with 2 to 3 ml of soft agar overlay (SAO) and overnight incubation at 37°C. All phages were purified by three consecutive rounds from single plaques. The plaques from the third round were then resuspended in SM buffer containing 0.1% (vol/vol) chloroform and stored at 4°C.

### Phage stock preparations.

In order to prepare a working phage stock with known PFU number, 40 ml of exponential growth indicator bacteria was infected with 100 μl of stored phage suspension, followed by incubation for 3 h at 37°C to allow the phages to multiply and increase in number. The broth was then centrifuged at 7,000 × *g* for 15 min and passed through a 0.45-μm-pore-size filter. Serial dilutions from the resultant broth were used in triplicate for plating in an SAO, as previously described, and the PFU/ml for each isolated phage was calculated.

### Transmission electron microscopy.

Purified phage particles in SM buffer were placed onto carbon-coated copper grids and negatively stained with 2% (wt/vol) uranyl acetate for 1 min. Particles were visualized using a FEI Tecnai G2 Spirit transmission electron microscope at an accelerating voltage of 80 kV at the Electron Microscopy Unit in Sheffield. Electron micrographs were recorded using a Gatan Orius 1000 digital camera and Digital Micrograph software. To observe the phages along with bacteria, 1 ml of exponentially growing cells was infected with phage at an MOI of 1 for 30 min, and, after centrifugation at 7,000 × *g* for 10 min, the pellet was resuspended with 1 ml of 3% glutaraldehyde for 1 h (room temperature) and examined by TEM as described above.

### Bacteriophage concentration by precipitation with polyethylene glycol (PEG 8000).

Further concentrations of phages were made in order to yield suitable working suspensions for protein profiling, genomic digestion, and DNA extraction. This was performed by precipitation with PEG 8000 (62). Briefly, 200 ml of exponential-phase indicator bacteria was infected with phage stock at an MOI of 0.01 for 3 h before 1 M NaCl was added during continuous mixing at 4°C for 1 h before any unlysed bacteria and cell debris were removed by centrifugation (5,000 × *g*). To precipitate the phage particles, 10% (wt/vol) PEG 8000 was gradually added with continuous mixing and left overnight at 4°C before centrifugation at 11,000 × *g* for 20 min to sediment precipitated phage. The resulting pellets were carefully resuspended overnight with 1 ml of SM buffer and stored at 4°C.

### Heat-inactivation of phage.

A phage suspension at 10^11^ PFU/ml was treated for 45 min at 80°C, which we established inactivated the virus with a 7-log-fold decrease in PFU/ml.

### One-step growth.

We used the procedure described previously (63), with some modifications. Briefly, 5-ml portions of exponentially growing cultures of E. faecalis OS16 were infected with SHEF2 phage at an MOI of 0.1. After 5 min of phage adsorption, bacteria were diluted 200 times (to prevent further infection) and then incubated at 37°C. Two samples were taken every 5 min, with one used to enumerate free phages in solution; the second was treated with 1% (vol/vol) chloroform to release intracellular phage and was used to enumerate the total phage number. Phage titers were determined as described previously, and plaques were counted on double-layer agar plates.

### Analysis of phage proteins.

To define the major proteins present in bacteriophages SHEF2, -4, -5, -6, and -7, SDS-PAGE was performed. A PEG 8000 concentrated phage stock was mixed with an equal volume of chloroform in order to release the phage particles before vortexing until an emulsion formed, followed by centrifugation at 10,000 × *g* for 10 min. Next, 50 μl from the upper layer (10^11^ to 10^13^ PFU/ml) was mixed with 50 μl of SDS-PAGE loading buffer, and the samples were heated at 95°C for 7 min. Then, 10 μl of lysate was loaded directly onto 4 to 12% NuPAGE Bis-Tris precast gels (Thermo Fisher Scientific, UK), and the samples were electrophoresed for 60 min. The gels were stained with InstantBlue (Expaedeon) and imaged using a gel documentation system (Image Scanner Power Look1120 USG; Amersham Bioscience).

### Mass spectrometry.

**(i) In-gel digestion.** Gel bands were excised and diced into 1-mm pieces prior to destaining using 200 mM ammonium bicarbonate (NH_4_HCO_3_) and 40% (vol/vol) acetonitrile (ACN). Samples were reduced and alkylated by the sequential addition of 10 mM dithiothreitol–50 mM NH_4_HCO_3_ at 50°C for 30 min and then 55 mM iodoacetamide at room temperature in the dark for 20 min. Gel pieces were dried using ACN before rehydration into a 10-ng/μl solution of trypsin prepared in 50 mM NH_4_HCO_3_. Samples were digested overnight at 37°C. Peptides in the supernatant were harvested and combined with peptides obtained by extraction of the gel pieces using 97% ACN and 0.1% (vol/vol) formic acid (FA). Peptides were dried by centrifugal evaporation using a Scanvac vacuum centrifuge (Labogene, Denmark) connected to a vacuum pump (Vacuubrand, Germany) before resuspension in 3% (vol/vol) ACN and 0.1% trifluoroacetic acid (TFA).

**(ii) LC-MS/MS.** Peptides were analyzed by nano-HPLC (UltiMate 3000 HPLC system; Thermo, Hemel Hempstead, UK) coupled to an amaZon ETD MS ion trap spectrometer (Bruker Daltonics, Bremen, Germany) using a nano-ESI spray. The nano-HPLC system and the ion trap spectrometer were controlled using Bruker Compass HyStar v3.2-SR2 software. The liquid chromatography system comprised a reversed-phase precolumn (LC Packings, Dionex) for sample desalting and a PepMap 100 reversed-phase C_18_ column (75 μm by 15 cm; Thermo) for peptide fractionation. The flow rate for precolumn loading was 30 μl/min of loading buffer (97% [vol/vol] ACN and 0.1% [vol/vol] TFA). Peptides were analyzed at a flow rate of 300 nl/min and separated by gradient elution using buffer A (3% [vol/vol] ACN and 0.1% [vol/vol] FA) and buffer B (97% ACN and 0.1% FA [vol/vol]) as follows: 4% buffer B (0 to 5 min), 5 to 38% buffer B (5 to 65 min), 38 to 90% buffer B (65 to 68 min), and 90% buffer B (68 to 73 min), followed by reequilibration at 4% buffer B. The electrospray was operated in positive-ion mode with a 4,500-V spray voltage, 10-lb/in^2^ gas pressure, and 150°C dry gas. The end plate offset of the mass spectrometer was set to −500 V and for the acquisition the standard method Proteomics Auto MSMS.

**(iii) Database searching.** Protein identifications were obtained using the Mascot software platform (MatrixScience, in-house server) to perform database searching against both the EMBOSS 6.5.7.0 and CDS annotation for the phage SHEF2 sequence (accession number MF678788). Contaminants, such as human skin keratin and trypsin, were assigned by parallel searching against the cRAP UniProt database (116 sequences, 38,459 residues; downloaded 30 January 2015). A concatenated target-decoy database search strategy was also used to estimate the false discovery rate. The tandem MS (MS/MS) search parameters were as follows: search enzyme, trypsin; maximum missed cleavages, 1; fixed modifications, carbamidomethyl (C); variable modifications, oxidation (M); mass values, monoisotopic; protein mass, unrestricted; peptide mass tolerance, ±1.2 Da; fragment mass tolerance, ±0.6 Da; and instrument type, ESI-TRAP.

### MLST designation of strains used.

To establish the MLST profile of each of the strains used, genomic DNA was extracted using a Wizard genomic DNA extraction kit (Promega), followed by PCR with previously described primers (64). The purified PCR fragments were sequenced in both directions by GATC Biotech AG using the same primers. Sequence type (ST) and cluster allelic profiling were determined by using the eBURST V3 software accessible via an internet-accessible database (http://efaecalis.mlst.net/) (65, 66). The MLST designations are presented in Table 3.

**TABLE 3:** Phage-host range of SHEF phages^*a*^

Strain/phage	Presence (+) or absence (–) of phage:					MLST
	SHEF2	SHEF4	SHEF5	SHEF6	SHEF7	
OS16	+	–	+	+	+	173
ER3/2s	+	–	–	+	+	21
EF1	–	–	+	–	–	34
EF2	–	+	–	–	–	283
EF3	–	+	–	–	–	97
EF54	+	–	–	+	+	381
OMGS3197	+	–	–	+	–	21
OMGS3198	+	–	–	–	–	55
OMGS3885	+	–	–	+	–	72
OMGS3919	+	–	–	+	+	97
OG1RF	+	–	–	+	+	1
V583	+	–	+	–	–	6
JH2-2	–	–	+	–	–	8
Total phages	9	2	4	7	5	

a A “+” indicates a zone of clearance in both spot and SAO screening tests, and a “–” indicates no evidence of clearance. The final column indicates the MLST designation established by sequencing according to http://efaecalis.mlst.net/.

### Phage DNA extraction.

To remove contaminating DNA and RNA from the PEG 8000 concentrated phage stocks, a 10-μg/ml DNase and RNase solution was added, followed by incubation at 37°C for 30 min before 100 μg/ml of proteinase K was added to degrade nucleases at 50°C for 45 min. The removal of proteins from nucleic acids was achieved by extraction with phenol-chloroform-isoamyl alcohol (25:24:1 [vol/vol]). DNA was precipitated by adding 2 volumes of ice-cold ethanol, followed by incubation overnight at –20°C before the DNA was pelleted at 16,000 × *g* for 20 min. The samples were then washed with 70% ethanol, before the DNA was dissolved in sterile Milli-Q water and stored at –20°C. These samples were used for nucleotide sequencing and RFLP analysis of phage genomes.

### RFLP analysis of phage genome size.

Phage genomic DNA was subjected to restriction digestion with HindIII, NdeI, and MfeI (New England Biolabs, UK) according to the manufacturer’s instructions. The digested products were separated by 1% ethidium bromide-agarose gel electrophoresis to determine the RFLP patterns, and gels were visualized using the Ingenius 3 Gel-Doc system (Syngene).

### Genome sequencing of phages SHEF2, SHEF4, and SHEF5.

Pure phage genomic DNA (20 μg) was sequenced by MicrobesNG on an Illumina MiSeq platform with 2 × 250-bp paired-end reads before identification of the closest available reference genome using Kraken (67), and reads were mapped using BWA mem (68) to assess the quality of the data. Reads were trimmed using Trimmomatic (69) before *de novo* assembly using SPAdes (70). An automated annotation was performed using Prokka (71), before the Mauve alignment software tool (72) was used to perform visual multiple comparisons, while multiple sequence alignment of individual genes was performed using Multalin (73). In addition, PHAST (PHAge Search Tool) (74) and PHASTER (PHAge Search Tool Enhanced Release) (75) web servers were used for further confirmation of annotated phage genomes. Conserved protein domains (where relevant) were detected using Pfam (76). Complete genomes were visualized using Artemis (77) and submitted to the National Center for Biotechnology Information as individual contigs under accession numbers MF678788, MF678789, and MF678790 for SHEF2, SHEF4, and SHEF5, respectively.

### Bioﬁlm assay on polystyrene plates.

The ability of the enterococcal strains to form a bioﬁlm on an abiotic surface was quantiﬁed based on a previously described method (78). Brieﬂy, E. faecalis strains were grown overnight in BHI at 37°C. The cultures were diluted 1:40 in fresh BHI medium, and 1 ml of this cell suspension was used to inoculate sterile, flat-bottomed 48-well polystyrene microtiter plates (Cellstar; Greiner Bio-One). Six wells per strain were inoculated with BHI alone as negative controls. These were then incubated under stationary conditions (aerobic) at 37°C for the times indicated (24 h in Fig. 5A or 6 days in Fig. S3). For longer-term biofilms, the medium was changed every 24 h. Broth was then carefully drawn off from wells, and 1 ml of fresh broth containing 10^8^ PFU/ml SHEF2 phage or BHI-only controls was added prior to incubation for another 3 h. Wells were then gently washed three times with 1 ml of PBS. The plates were inverted on a paper towel, air dried, and stained with 1% crystal violet for 15 min. The wells were washed again three times, the crystal violet was solubilized in 500 μl of ethanol-acetone (80:20, vol/vol), and the OD_570_ was measured using a microplate reader (Tecan Infinite 200; Tecan, Austria). Each assay was performed in triplicate and repeated three times.

### Adsorption rate experiments.

Adsorption rate experiments were performed according to a previously described procedure (79), with small modifications. Briefly, overnight cultures of the bacterial strains were diluted 1:100 in BHI medium. When the OD_600_ value of the reference strains reached 2.0, 1 ml of culture was diluted 10-fold in fresh BHI (5 × 10^8^ CFU/ml). Phages were added at an MOI of 1 to the diluted culture, mixed gently, and incubated at 37°C. Incubation was continued for 12 min, with samples (100 μl) collected at 1-min intervals, and samples were diluted immediately in 900 μl of cooled SM buffer. The diluted samples were centrifuged at 10,000 × *g* (4°C) for 5 min and passed through a 0.45-μm-pore-size filter. Finally, the titers of unabsorbed phages in the supernatant were determined after serial dilution. The adsorption levels are represented as the percentage of the total number of phages, calculated as follows: [(initial phage titer/free phage titer in supernatant)/initial phage titer] × 100.

### *In vitro* bioﬁlm assay on tooth root surface.

The ability of SHEF2 phage to eradicate E. faecalis biofilm on extracted natural root surfaces was quantiﬁed based on a previously described method using resazurin dye change (80, 81). To produce a growth surface, 1-mm-thick root slices were cross-sectioned above the root bifurcation area of multirooted human teeth (ethical approval number STH18841) by a water-cooled 0.1-mm cutting saw (MEDREK 859088) and divided into two groups in 24-well microtiter plates (Cellstar) with final equal surface area for each group. At day 7 (168 h), an E. faecalis biofilm was grown as previously described, i.e., using 24-h medium changes. After this, the first group (treated) was treated with 1 ml of BHI containing 10^8^ PFU/ml SHEF2 phage with BHI only used as a control (untreated) before incubation for another 3 h. The root slices were transferred into 48-well microtiter plates and washed three times with 1 ml of PBS. After treatment and washing, 0.350 ml of PBS containing resazurin solution at a final concentration of 1 μg/ml was added to both groups in a 48-well plate, followed by incubation at 37°C for 20 min. Then, 0.3 ml from each well of both groups (treated and untreated) was transferred to 96-well microtiter plates, and the ﬂuorescence was read by using a microplate spectrofluorometer (Tecan Infinite 200; settings, λ_ex_ 570 nm and λ_em_ 590 nm). The reading for the nonemitting resazurin dye (treated) was subtracted from a control group that contained only resazurin dye. This assay was repeated twice, with at least three samples in each group. In addition, we performed a standard curve of bacteria (CFU/ml) versus resazurin (Fig. S3C).

### Zebrafish as *in vivo* model for phage treatment.

Zebrafish maintenance and experimental work were performed in accordance with UK Home Office regulations and UK Animals (Scientific Procedures) Act 1986. Ethical approval was given by the University of Sheffield Local Ethical Review Panel. Wild-type inbred zebrafish larvae were obtained from The Bateson Centre, University of Sheffield. All larvae were maintained in E3 medium at 30°C according to standard protocols and monitored for up to 4 days postfertilization. Groups of at least 20 larvae were used for each treatment condition. Tricaine-anesthetized embryos were injected individually with 2 nl of E. faecalis (30,000 CFU) into the Ducts of Cuvier of dechorionated embryos at 30 h postfertilization, and larvae were incubated for 2 h before injection with 2 nl of SHEF2 phage (MOI of 20, in relation to the original bacterial inoculum) or PBS. Fish health status was monitored for up to 72 hpi. In parallel, larvae were also injected with PBS only, E. faecalis only, or phage only alongside heat-killed phage. In all cases, fish health status was monitored for up to 72 hpi. Zebrafish mortality was assessed based on the examination of the presence of a heart beat and blood circulation (82). Images of 10% formalin-fixed zebrafish larvae were captured using a fluorescence zoom microscope (Axio Zoom.V16; Zeiss) with Zen Black software.

### Assessment of phage adhesion.

Phage adsorption rates were established as described earlier, with free phage particles of SHEF2 counted at 0 min, 10 min, and 24 hpi with *epaB* mutant cells and compared to control OG1RF cells. The free phage particles were also counted from a 24-h suspension after treatment with 1% (vol/vol) chloroform in order to lyse all cells to release any possible trapped intracellular phage. In addition, 1-ml samples from the 24-h phage-bacterium suspension were pelleted and resuspended in ice-cold 1 ml of PBS in 0.28 M NaCl (previously shown to break down electrostatic interactions between phage and cell wall material [62]) for 10 min at 4°C. These samples were then passed through a 0.45-μm-pore-size filter to collect released phage, and the titers of free phages in the supernatant were determined. The phage count from the 0.28 M NaCl sample was deducted from the free phage count of the PBS control group (0.150 M NaCl) to assess how many were adsorbed and how many were released by the increased NaCl. In addition, and to exclude cell lysis caused by increasing the molarity of NaCl, the CFU were assessed and compared to the control group. This experiment was repeated twice in triplicate each time.

### Statistical analysis.

Statistical analysis was performed with a *t* test using Prism v7.0 (GraphPad, La Jolla, CA), and statistical significance was assumed if the *P* value was <0.05. For zebrafish experiments, Kaplan-Meier survival curves were compared using a log rank test, and differences between unhealthy groups were evaluated using one-way analysis of variance (ANOVA).

## Supplementary Material

Supplemental file 1
