# Supplementary material for: Identification of Novel Bacteriophages with Therapeutic Potential That Target Enterococcus faecalis
Source: Infect Immun. 2019 Oct 18;87(11):e00512-19. doi: 10.1128/IAI.00512-19 (PMC6803325; doi:10.1128/IAI.00512-19)
Supplement: Supplemental file 1 [file IAI.00512-19-s0001.pdf]

Figure S1

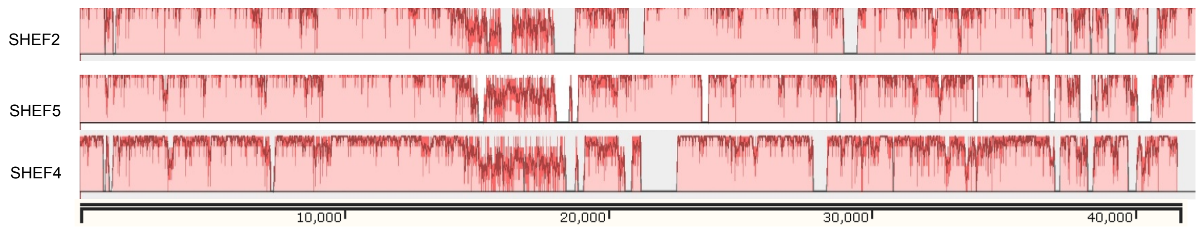

Figure S2

A.

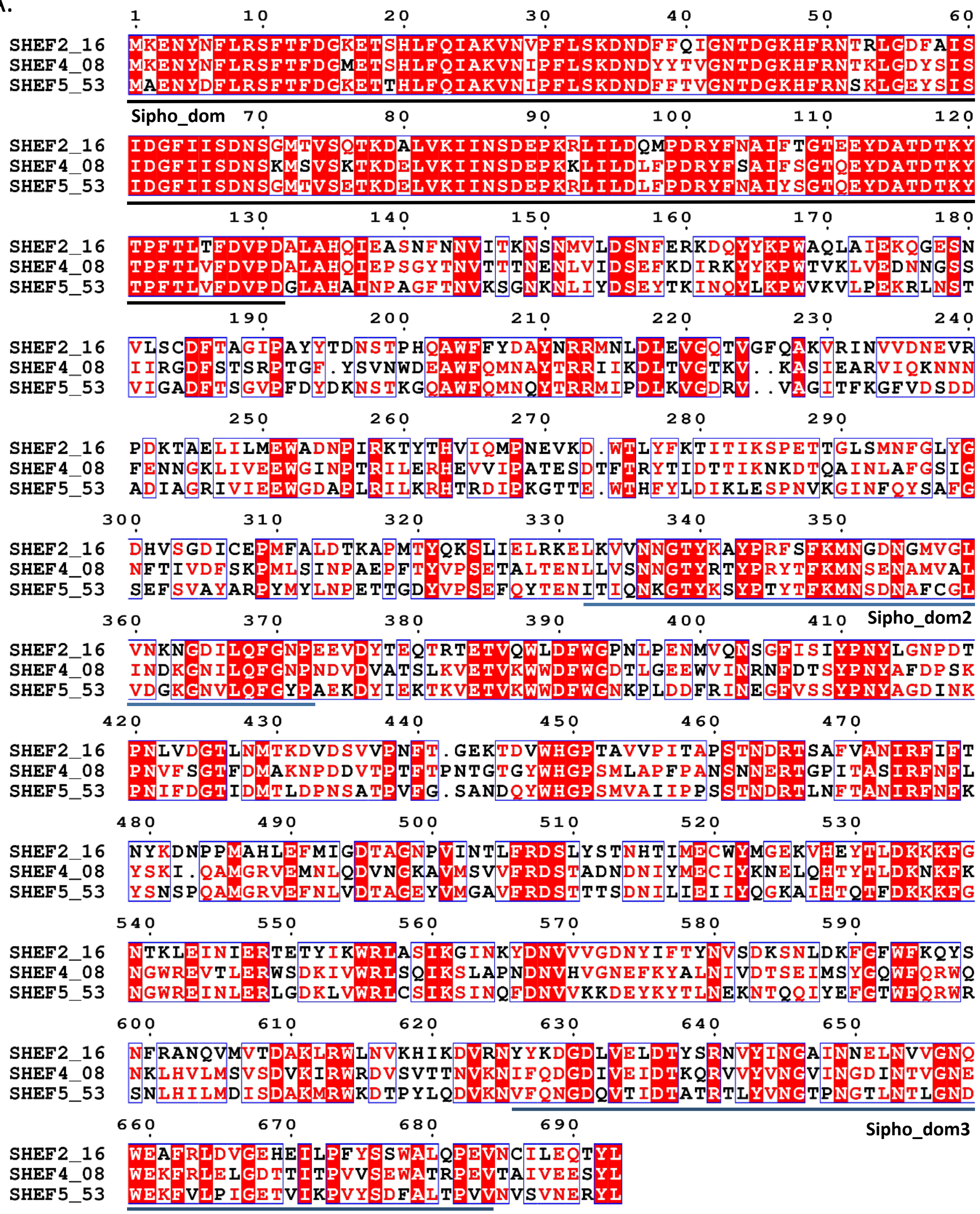

SHEF2_16 MKENYNFLRSFTFDGKETSHLFQIAKVNVPFLSKDNDFFQIGNTDGKHFRNTRLGDFAIS
SHEF4_08 MKENYNFLRSFTFDGMETSHLFQIAKVNIPFLSKDNDYYTVGNTDGKHFRNTKLGDYSIS
SHEF5_53 MAENYDFLRSFTFDGKETTHLFQIAKVNIPFLSKDNDFFTVGNTDGKHFRNSKLGEYSIS
Sipho_dom
SHEF2_16 IDGFIISDNSGMTVSQTKDALVKIINSDEPKRLILDQMPDRYFNAIFTGTEEYDATDTKY
SHEF4_08 IDGFIISDNSKMSVSKTKDELVKIINSDEPKKLILDLFPDRYFSAIFSGTQEYDATDTKY
SHEF5_53 IDGFIISDNSGMTVSETKDELVKIINSDEPKRLILDLFPDRYFNAIYSGTQEYDATDTKY
SHEF2_16 TPFTLTFDVPDALAHQIEASNFNNVITKNSNMVLDSNFERKDQYYKPWAQLAIEKQGESN
SHEF4_08 TPFTLVFDVPDALAHQIEPSGYTNVTTTNENLVIDSEFKDIRKYYKPWTVKLVEDNNGSS
SHEF5_53 TPFTLVFDVPDGLAHAINPAGFTNVKSGNKNLIYDSEYTKINQYLKPWVKVLPEKRLNST
SHEF2_16 VLSCDFTAGIPAYYTDNSTPHQAWFFYDAYNRRMNLDLEVGQTVGFQAKVRINVVDNEVR
SHEF4_08 IIRGDFSTSRPTGF.YSVNWDEAWFQMNAYTRRIIKDLTVGTKV..KASIEARVIQKNNN
SHEF5_53 VIGADFTSGVPFDYDKNSTKGQAWFQMNQYTRRMIPDLKVGDRV..VAGITFKGFVDSDD
SHEF2_16 PDKTAELILMEWADNPIRKTYTHVIQMPNEVKD.WTLYFKTITIKSPETTGLSMNFGLYG
SHEF4_08 FENNGKLIVEEWGINPTRILERHEVVIPATESDTFTRYTIDTTIKNKDTQAINLAFGSIG
SHEF5_53 ADIAGRIVIEEWGDAPLRILKRHTRDIPKGTTE.WTHFYLDIKLESPNVKGINFQYSAFG
SHEF2_16 DHVSGDICEPMFALDTKAPMTYQKSLIELRKELKVVNNGTYKAYPRFSFKMNGDNGMVGL
SHEF4_08 NFTIVDFSKPMLSINPAEPFTYVPSETALTENLLVSNNGTYRTYPRYTFKMNSENAMVAL
SHEF5_53 SEFSVAYARPYMYLNPETTGDYVPSEFQYTENITIQNKGTYKSYPTYTFKMNSDNAFCGL
Sipho_dom2
SHEF2_16 VNKNGDILQFGNPEEVDYTEQTRTETVQWLDFWGPNLPENMVQNSGFISIYPNYLGNPDT
SHEF4_08 INDKGNILQFGNPNDVDVATSLKVETVKWWDFWGDTLGEEWVINRNFDTSYPNYAFDPSK
SHEF5_53 VDGKGNVLQFGYPAEKDYIEKTKVETVKWWDFWGNKPLDDFRINEGFVSSYPNYAGDINK
SHEF2_16 PNLVDGTLNMTKDVDSVVPNFT.GEKTDVWHGPTAVVPITAPSTNDRTSAFVANIRFIFT
SHEF4_08 PNVFSGTFDMAKNPDDVTPTFTPNTGTGYWHGPSMLAPFPANSNNERTGPITASIRFNFL
SHEF5_53 PNIFDGTIDMTLDPNSATPVFG.SANDQYWHGPSMVAIIPPSSTNDRTLNFTANIRFNFK
SHEF2_16 NYKDNPPMAHLEFMIGDTAGNPVINTLFRDSLYSTNHTIMECWYMGEKVHEYTLDKKKFG
SHEF4_08 YSKI.QAMGRVEMNLQDVNGKAVMSVVFRDSTADNDNIYMECIYKNELQHTYTLDKNKFK
SHEF5_53 YSNSPQAMGRVEFNLVDTAGEYVMGAVFRDSTTTSDNILIEIIYQGKAIHTQTFDKKKFG
SHEF2_16 NTKLEINIERTETYIKWRLASIKGINKYDNVVVGDNYIFTYNVSDKSNLDKFGFWFKQYS
SHEF4_08 NGWREVTLERWSDKIVWRLSQIKSLAPNDNVHVGNEFKYALNIVDTSEIMSYGQWFQRWQ
SHEF5_53 NGWREINLERLGDKLVWRLCSIKSINQFDNVVKKDEYKYTLNEKNTQQIYEFGTWFQRWR
SHEF2_16 NFRANQVMVTDAKLRWLNVKHIKDVRNYYKDGDLVELDTYSRNVYINGAINNELNVVGNQ
SHEF4_08 NKLHVLMSVSDVKIRWRDVSVTTNVKNIFQDGDIVEIDTKQRVVYVNGVINGDINTVGNE
SHEF5_53 SNLHILMDISDAKMRWKDTPYLQDVKNVFQNGDQVTIDTATRTLYVNGTPNGTLNTLGND
Sipho_dom3
SHEF2_16 WEAFRLDVGEHEILPFYSSWALQPEVNCILEQTYL
SHEF4_08 WEKFRLELGDTTITPVVSEWATRPEVTAIVEESYL
SHEF5_53 WEKFVLPIGETVIKPVYSDFALTPVVNVSVNERYL

B.

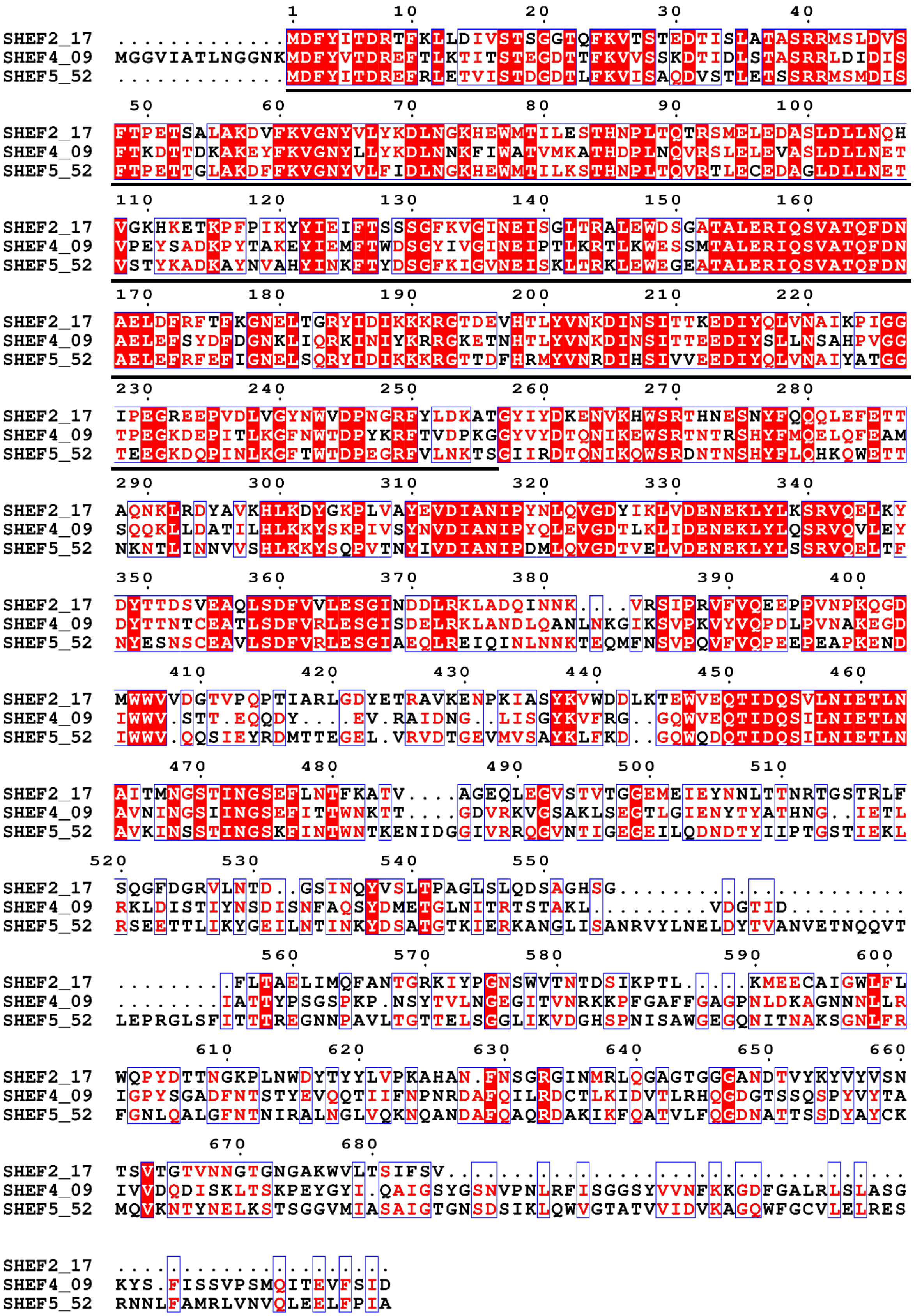
1 10 20 30 40
SHEF2_17 .............MDFYITDRTFKLLDIVSTSGGTQFKVTSTEDTISLATASRRMSLDVS
SHEF4_09 MGGVIATLNGGNKMDFYVTDREFTLKTITSTEGDTTFKVVSSKDTIDLSTASRRLDIDIS
SHEF5_52 .............MDFYITDREFRLETVISTDGDTLFKVISAQDVSTLETSSRRMSMDIS
50 60 70 80 90 100
SHEF2_17 FTPETSALAKDVFKVGNYVLYKDLNGKHEWMTILESTHNPLTQTRSMELEDASLDLLNQH
SHEF4_09 FTKDTTDKAKEYFKVGNYLLYKDLNNKFIWATVMKATHDPLNQVRSLELEVASLDLLNET
SHEF5_52 FTPETTGLAKDFFKVGNYVLFIDLNGKHEWMTILKSTHNPLTQVRTLECEDAGLDLLNET
110 120 130 140 150 160
SHEF2_17 VGKHKETKPFPIKYYIEIFTSSSGFKVGINEISGLTRALEWDSGATALERIQSVATQFDN
SHEF4_09 VPEYSADKPYTAKEYIEMFTWDSGYIVGINEIPTLKRTLKWESSMTALERIQSVATQFDN
SHEF5_52 VSTYKADKAYNVAHYINKFTYDSGFKIGVNEISKLTRKLEWEGEATALERIQSVATQFDN
170 180 190 200 210 220
SHEF2_17 AELDFRFTFKGNELTGRYIDIKKKRGTDEVHTLYVNKDINSITTKEDIYQLVNAIKPIGG
SHEF4_09 AELEFSYDFDGNKLIQRKINIYKRRGKETNHTLYVNKDINSITTEEDIYSLLNSAHPVGG
SHEF5_52 AELEFRFEFIGNELSQRYIDIKKKRGTTDFHRMYVNRDIHSIVVEEDIYQLVNAIYATGG
230 240 250 260 270 280
SHEF2_17 IPEGREEPVDLVGYNWVDPNGRFYLDKATGYIYDKENVKHWSRTHNESNYFQQQLEFETT
SHEF4_09 TPEGKDEPITLKGFNWTDPYKRFTVDPKGGYVYDTQNIKEWSRTNTRSHYFMQELQFEAM
SHEF5_52 TEEGKDQPINLKGFTWTDPEGRFVLNKTSGIIRDTQNIKQWSRDNTNSHYFLQHKQWETT
290 300 310 320 330 340
SHEF2_17 AQNKLRDYAVKHLKDYGKPLVAYEVDIANIPYNLQVGDYIKLVDENEKLYLKSRVQELKY
SHEF4_09 SQQKLLDATILHLKKYSKPIVSYNVDIANIPYQLEVGDTLKLIDENEKLYLQSRVQVLEY
SHEF5_52 NKNTLINNVVSHLKKYSQPVTNYIVDIANIPDMLQVGDTVELVDENEKLYLSSRVQELTF
350 360 370 380 390 400
SHEF2_17 DYTTDSVEAQLSDFVVLESGINDDLRKLADQINNK....VRSIPRVFVQEEPPVNPKQGD
SHEF4_09 DYTTNTCEATLSDFVRLESGISDELRKLANDLQANLNKGIKSVPKVYVQPDLPVNAKEGD
SHEF5_52 NYESNSCEAVLSDFVRLESGIAEQLREIQINLNNKTEQMFNSVPQVFVQPEEPEAPKEND
410 420 430 440 450 460
SHEF2_17 MWWVVDGTVPQPTIARLGDYETRAVKENPKIASYKVWDDLKTEWVEQTIDQSVLNIETLN
SHEF4_09 IWWV.STT.EQQDY....EV.RAIDNG..LISGYKVFRG..GQWVEQTIDQSILNIETLN
SHEF5_52 IWWV.QQSIEYRDMTTEGEL.VRVDTGEVMVSAYKLFKD..GQWQDQTIDQSILNIETLN
470 480 490 500 510
SHEF2_17 AITMNGSTINGSEFLNTFKATV....AGEQLEGVSTVTGGEMEIEYNNLTTNRTGSTRLF
SHEF4_09 AVNINGSIINGSEFITTWNKTT....GDVRKVGSAKLSEGTLGIENYTYATHNG..IETL
SHEF5_52 AVKINSSTINGSKFINTWNTKENIDGGIVRRQGVNTIGEGEILQDNDTYIIPTGSTIEKL
520 530 540 550
SHEF2_17 SQGFDGRVLNTD..GSINQYVSLTPAGLSLQDSAGHSG......................
SHEF4_09 RKLDISTIYNSDISNFAQSYDMETGLNITRTSTAKL.........VDGTID.........
SHEF5_52 RSEETTLIKYGEILNTINKYDSATGTKIERKANGLISANRVYLNELDYTVANVETNQQVT
560 570 580 590 600
SHEF2_17 .........FLTAELIMQFANTGRKIYPGNSWVTNTDSIKPTL.....KMEECAIGWLFL
SHEF4_09 ........IATTYPSGSPKP.NSYTVLNGEGITVNRKKPFGAFFGAGPNLDKAGNNNLLR
SHEF5_52 LEPRGLSFITTTREGNNPAVLTGTTELSGGLIKVDGHSPNISAWGEGQNITNAKSGNLFR
610 620 630 640 650 660
SHEF2_17 WQPYDTTNGKPLNWDYTYYLVPKAHAN.FNSGRGINMRLQGAGTGGGANDTVYKYVYVSN
SHEF4_09 IGPYSGADFNTSTYEVQQTIIFNPNRDAFQILRDCTLKIDVTLRHQGDGTSSQSPYVYTA
SHEF5_52 FGNLQALGFNTNIRALNGLVQKNQANDAFQAQRDAKIKFQATVLFQGDNATTSSDYAYCK
670 680
SHEF2_17 TSVTGTVNNGTGNGAKWVLTSIFSV...................................
SHEF4_09 IVVDQDISKLTSKPEYGYI.QAIGSYGSNVPNLRFISGGSYVVNFKKGDFGALRLSLASG
SHEF5_52 MQVKNTYNELKSTSGGVMIASAIGTGNSDSIKLQWVGTATVVIDVKAGQWFGCVLELRES
SHEF2_17 ....
SHEF4_09 KYS.FISSVPSMQITEVFSID
SHEF5_52 RNNLFAMRLVNVQLEELFPIA

Figure S3

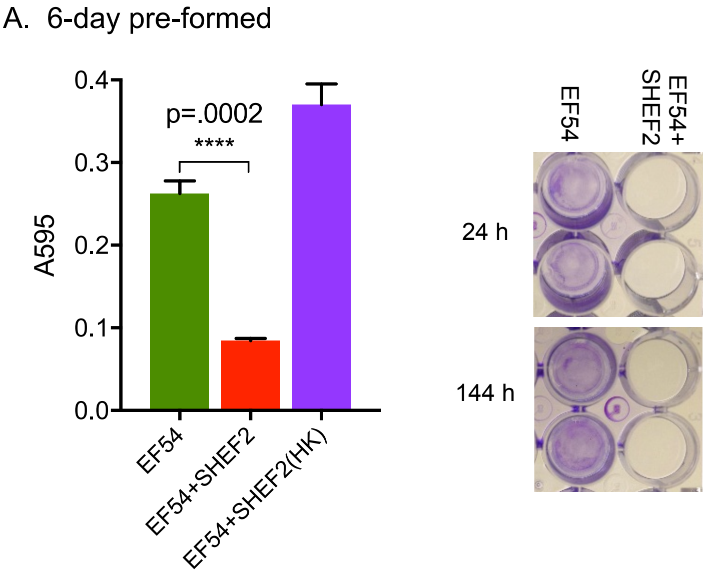
A. 6-day pre-formed
p=.0002
****
A595
0.4
0.3
0.2
0.1
0.0
EF54
EF54+SHEF2
EF54+SHEF2(HK)
EF54
EF54+
SHEF2
24 h
144 h

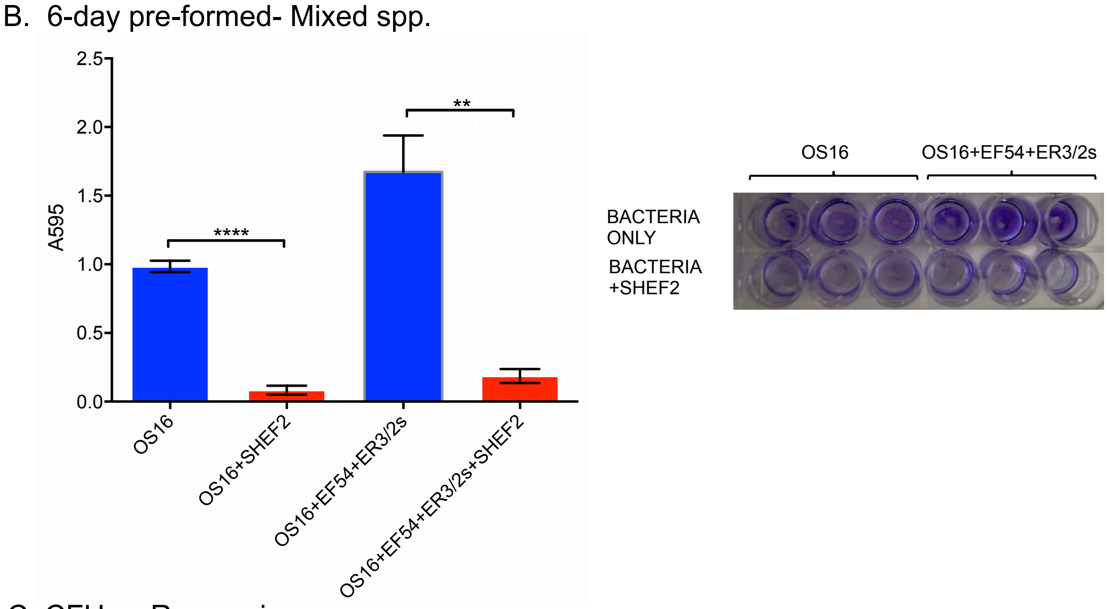
B. 6-day pre-formed- Mixed spp.
2.5
2.0
1.5
1.0
0.5
0.0
A595
****
**
OS16
OS16+SHEF2
OS16+EF54+ER3/2s
OS16+EF54+ER3/2s+SHEF2
OS16
OS16+EF54+ER3/2s
BACTERIA
ONLY
BACTERIA
+SHEF2

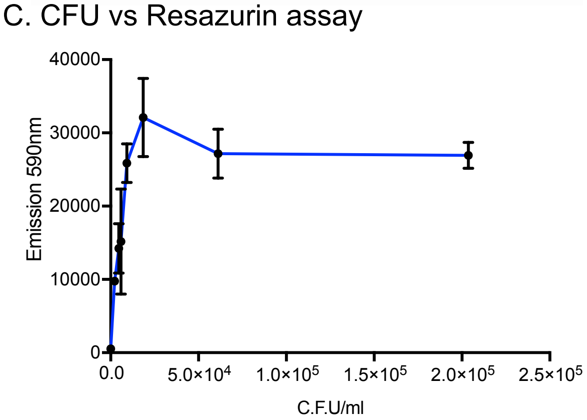
C. CFU vs Resazurin assay
40000
30000
20000
10000
0
Emission 590nm
0.0
5.0×10^4
1.0×10^5
1.5×10^5
2.0×10^5
2.5×10^5
C.F.U/ml

Figure S4

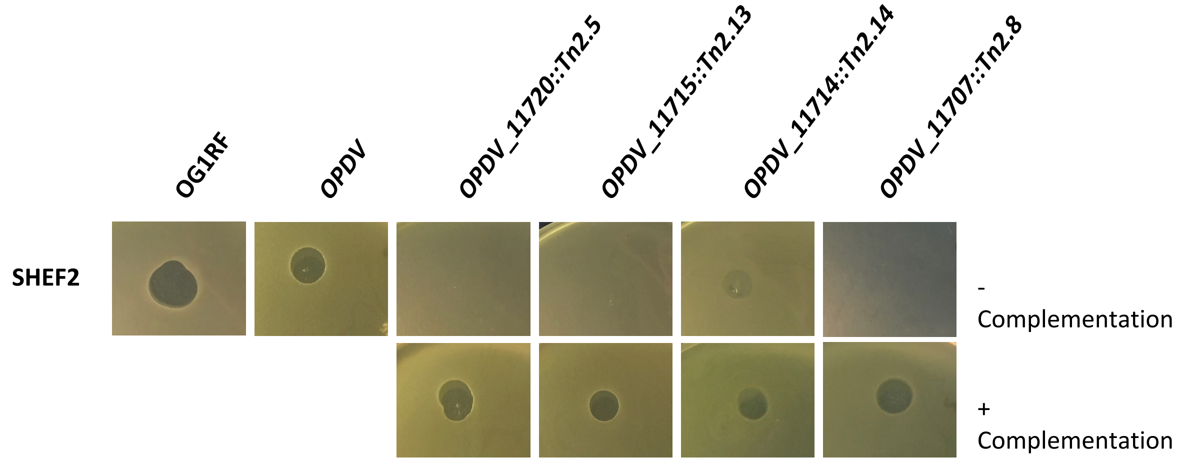
OG1RF
OPDV
OPDV_11720::Tn2.5
OPDV_11715::Tn2.13
OPDV_11714::Tn2.14
OPDV_11707::Tn2.8
SHEF2
- Complementation
+ Complementation

Figure S5

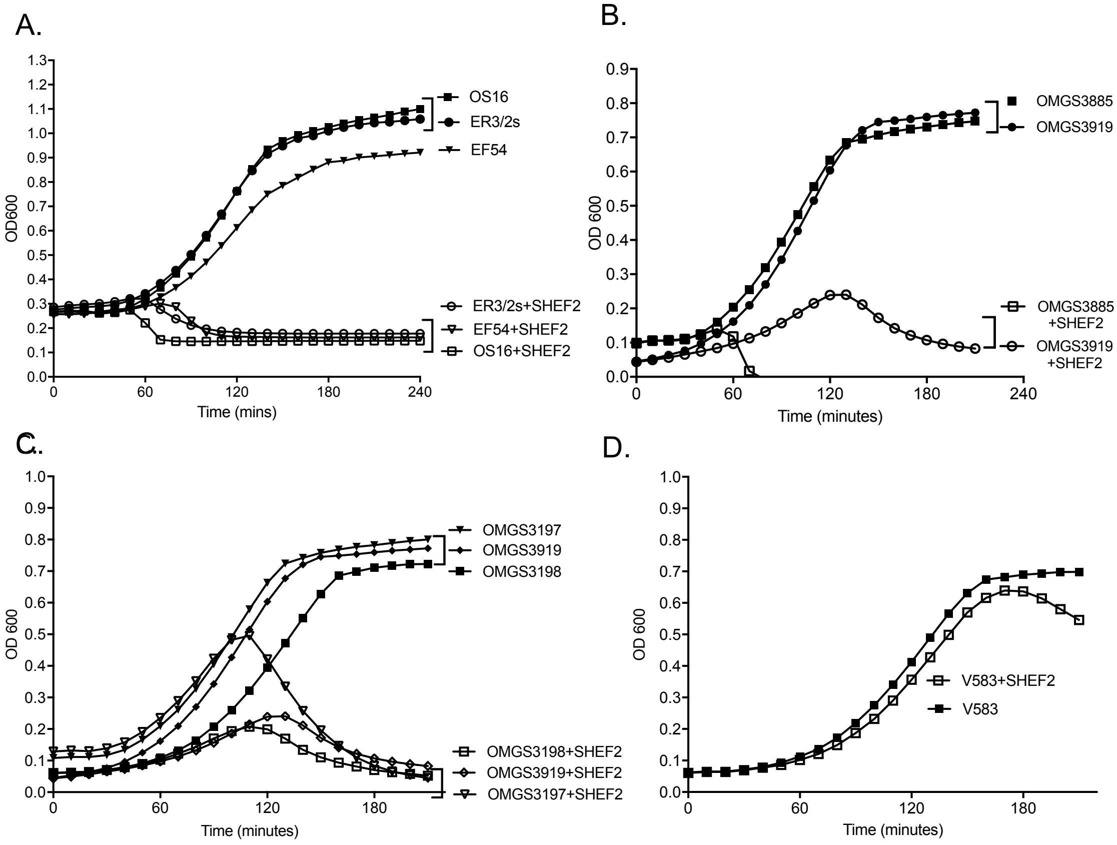
A.
OD600
Time (mins)
OS16
ER3/2s
EF54
ER3/2s+SHEF2
EF54+SHEF2
OS16+SHEF2
B.
OD 600
Time (minutes)
OMGS3885
OMGS3919
OMGS3885 +SHEF2
OMGS3919 +SHEF2
C.
OD 600
Time (minutes)
OMGS3197
OMGS3919
OMGS3198
OMGS3198+SHEF2
OMGS3919+SHEF2
OMGS3197+SHEF2
D.
OD 600
Time (minutes)
V583+SHEF2
V583

SUPPLEMENTARY FIGURE LEGENDS

Figure S1 Mauvealign image of the entire genome of SHEF 2,4,5.

Figure S2. Multiple sequence alignment of SHEF minor tail genes produced by Multalin. A) SHEF2_16, 4_08 and 5_53; with conserved domains underlined in black and blue. B) SHEF2_17, 4_09 and 5_52; the phage anti-receptor (TIGR01665) and smaller domain of phage tail endopeptidase (pfam06605) are underlined

Figure S3. **Biofilm growth and affects of SHEF2 addition**
A. Biofilms were grown on Polystyrene surfaces for 6-days before addition of either live or heat-killed SHEF2. Bar charts (left) represent data from 6 wells per condition with A570 quantified after extraction of Crystal violet. Samples treated with live phage are labelled: +SHEF2; while those with heat-killed: +SHEF2 (HK); strain names are as elsewhere. Mean with SD and students t-test to compare conditions ($P<0.0001$). Images of wells are shown to illustrate these data at both 24 and 144h.
B. Mixed species biofilms were grown under identical conditions as S3A. Bar charts display A570 after Crystal Violet staining. Mean of 6-wells with SD is shown with used students t-test to compare conditions ($P<0.0001$). (right) an example image of microtitre plate is shown after CV staining.
C. Triplicate cultures of strain EF54 Were grown overnight and then diluted to the cell numbers shown and incubated with resazurin under identical conditions to the assay in Fig. 5. Emission at 590nm is shown with SD of mean readings.

S4- Infection of OG1RF strains with SHEF2. Double layer spot-plating assays of strains infected with SHEF2 ($1x10^7$ PFU) were performed and incubated o/n before imaging.

S5. Sensitivity assays. SHEF2 Phage ($1x10^7$ PFU) was added to 100ml of an overnight culture of strains indicated diluted to OD600 0.1 ($1x10^8$ cfu/ml) in fresh warmed BHI broth. 150μl was then placed in 96-well plates and measured with shaking over time with OD measured at the points indicated.
